# Supplementary material for: High-Intensity Pulse Magnetic Fields Affect Redox Homeostasis and Survival Rate of Escherichia coli According to Initial Level of Intracellular Glucose
Source: Biomolecules. 2025 Nov 5;15(11):1550. doi: 10.3390/biom15111550 (PMC12649941; doi:10.3390/biom15111550)
Supplement: Supplementary file 1 [file biomolecules-15-01550-s001.zip › supplementary.pdf]

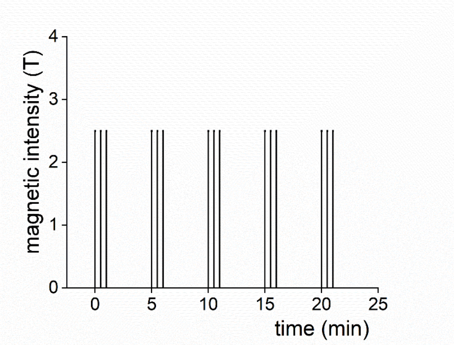

Figure S1 The pulse mode of HI-OMF used in this study. Each black line represents a sinusoidal magnetic field pulse that lasts for 10ms and has a maximum intensity of 2.5T.

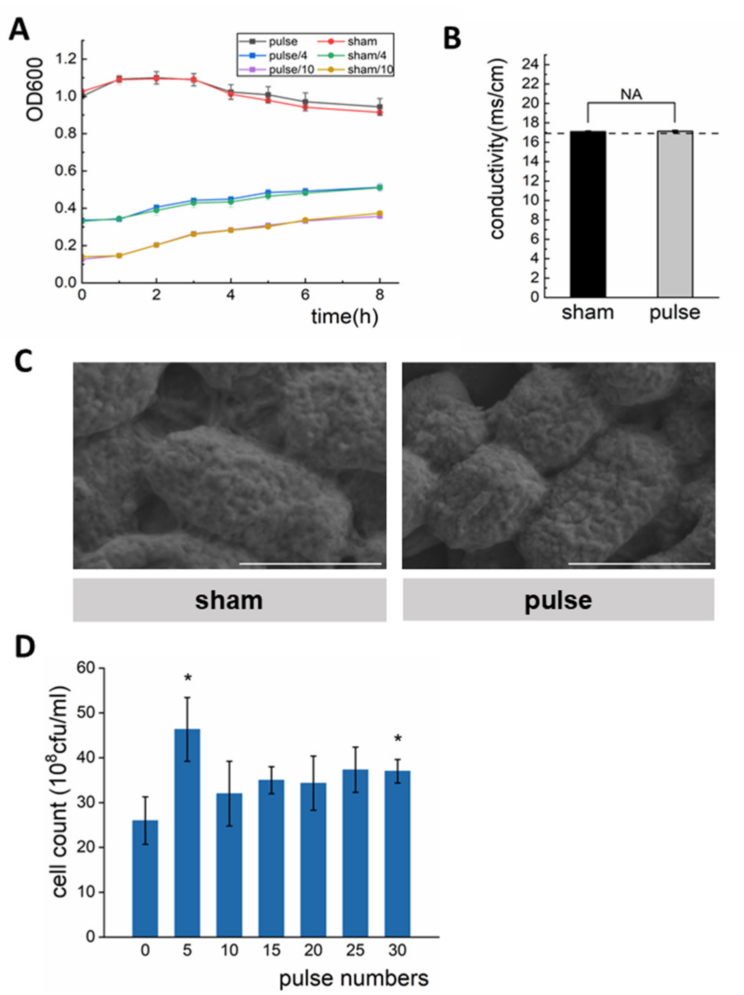

Figure S2 Effects of HI-PEMF on growth (**A**), suspension conductivity (**B**), and morphological (**C**) of *E. coli* in the stable phase. **D.** Cell count in Fig. 1f. Sham, sham group; Pulse, HI-PEMF treatment group; Sham/4 and pulse/4, re-culturing respectively of sham group and HI-PEMF treatment group after a fourfold dilution of the bacterial concentration; Sham/10 and pulse/10, re-culturing respectively of sham group and HI-PEMF treatment group after a tenfold dilution of the bacterial concentration. Scar bar, 1 μ m. Data were all expressed as mean ± SD (n = 3). NA, no significant difference

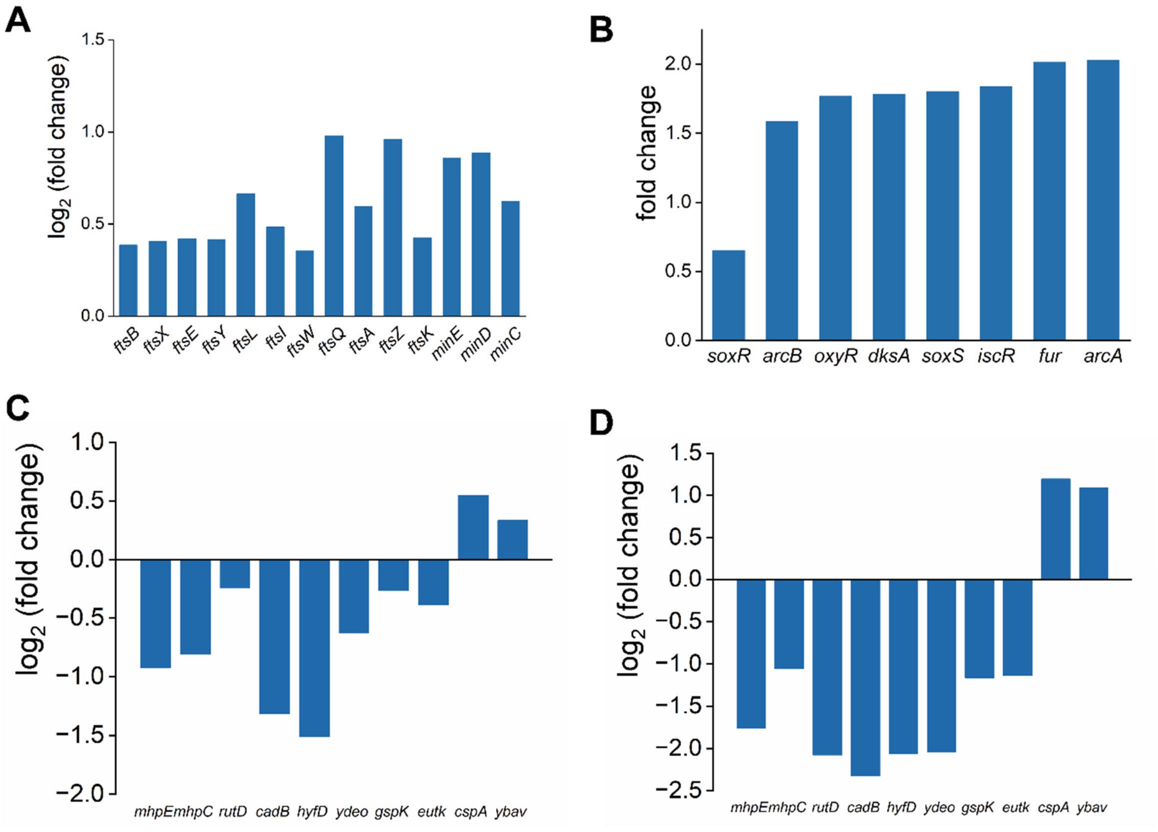

Figure S3 The fold changes of genes in *E. coli* cells after HI-PMF treatment. **A.** The fold changes of genes related to division in *E. coli* cells after HI-PMF treatment. **B.** The fold changes of genes related to antioxidant in *E. coli* after HI-PMF treatment. **C, D.** the gene fold changes calculated by qPCR (**C**) and transcriptome analyses (**D**). The data represents the average of three parallel samples (**C**) or two parallel samples (**A, B** and **D**).

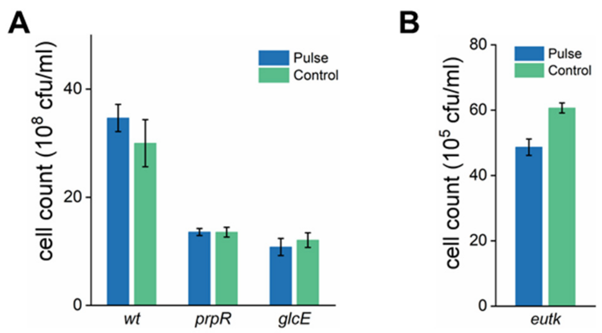

Figure S4 Cell count of wt, *prpR* overexpression strain, *glcE* overexpression strain, and *eutk* overexpression strain. **A.** Cell count of *wt*, *prpR* overexpression strain, and *glcE* overexpression strain in Fig. 3d. **B.** Cell count of *eutk* overexpression strain in Fig. 3d. Data were all expressed as mean ± SD (n = 3).

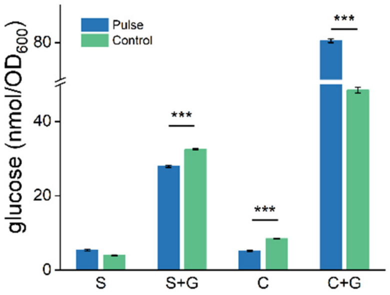

Figure S5 The intracellular glucose concentration of the four groups with/without HI-PMF treatment.

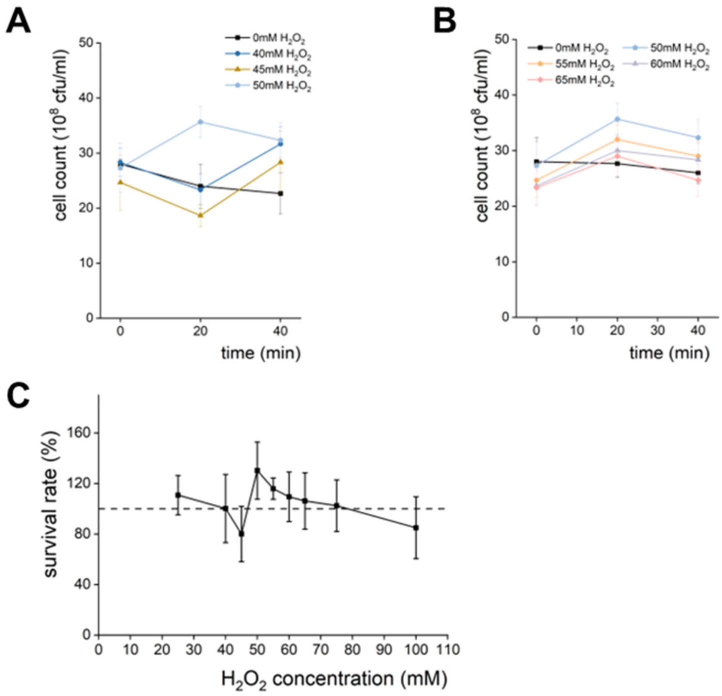

Figure S6 The changes of cell count and survival rates of *E. coli* with H_2O_2. **A.** The changes of cell count of *E. coli* treated with 40mM, 45mM and 50mM H_2O_2. **B.** The changes of cell count of *E. coli* treated with 50mM, 55mM, 60mM H_2O_2 and 65 mM H_2O_2. **C.** The survival rates measured 20 minutes after treatment across the H_2O_2 gradient. Data were all expressed as mean ± SD (n = 3).

Table S1. Bacterial strains used in this study.

Strain	Genotype	Source
ct	*E. coli* BL21 (DE3) Δ*HO396_18235* :: SoNar	This study
Δ*pgm*	ct Δ*pgm*	This study
Δ*kilR*	ct Δ*kilR*	This study
Δ*soxR*	ct Δ*soxR*	This study
soxR	ct P_{T7}:*soxR*	This study
prpR	ct P_{T7}:*prpR*	This study
glcE	ct P_{T7}: *glcE*	This study
eutK	ct P_{T7}: *eutK*	This study

Table S2. The primers used in this study.

Primer	Sequence
sgRNA1-Forward	CTGGATTACATTCGTGATGGGTTTTAGAGCTAGAAATAGC
sgRNA1-Reverse	CCATCACGAATGTAATCCAGACTAGTATTATACCTAGGAC
Fs1-Forward	GGTGCGAAACCGTAACCC
Fs1-Reverse	AGTGAGTCGTATTAATTTGTCGGCGTCCTGCTC
Rs1-Forward	TGAGGGGTTTTTGCTGGTTATCGCACGCAGACCA
Rs1-Reverse	TCCCGTTAATGCAGCAAA
sgRNA2-Forward	GATGAATCCGAACCACTACCGTTTTAGAGCTAGAAATAGC
sgRNA2-Reverse	GGTAGTGGTTCGGATTCATCACTAGTATTATACCTAGGAC
Fspg-Forward	AAGGTTTGCGGAACTATCT
Fspg-Reverse	ATAGAAGCACCGTTGCCGGGTTATGGGACGGTGTA
Rspg-Forward	GGCAACGGTGCTTCTAT
Rspg-Reverse	TTGGTCTTCATTGGGAT
sgRNA3-Forward	TTGCCGAGGCAATATATGTCGTTTTAGAGCTAGAAATAGC
sgRNA3-Reverse	GACATATATTGCCTCGGCAAACTAGTATTATACCTAGGAC
Fski-Forward	GTAGTTACAACAGTGGCTT
Fski-Reverse	TTATGATTGCACATCACGGTTTACCTGTAAAGGC
Rski-Forward	GTGATGTGCAATCATAA
Rski-Reverse	GAGTCGGTCTTGTCTGT
sgRNA4-Forward	CGTTTGGCGTGTTGCCCGAAGTTTTAGAGCTAGAAATAGC
sgRNA4-Reverse	TTCGGGCAACACGCCAAACGACTAGTATTATACCTAGGAC
Fsso-Forward	GCCGCCGCAGGTGTTTAT
Fsso-Reverse	AAATCGCTTTACCTCAAGT
Rsso-Forward	ACTTGAGGTAAAGCGATTTTGGAGGTTCCGGTTGGTGT
Rsso-Reverse	CGGACAATCCCCTGCCA
Pr-Forward	CGGGATCCATGGCACATCCACCACGGC
Pr-Reverse	ACGCGTCGACTCAGCTTTTCAGCCGCC
Gl-Forward	CGAGCTCATGCTACGCGAGTGTGATT
Gl-Reverse	CCCAAGCTTTCAAAGTTCCGCGTACAT
Eu-Forward	CGGGATCCATGATCAATGCACTGGGA
Eu-Reverse	CCCAAGCTTTTAATGGGGCTTGAGACG
So-Forward	CGGGATCCATGGAAAAGAAATTACCCCGCA
So-Reverse	CCCAAGCTTTTAGTTTTGTTCATCTTCCA
mhpE1	GCCATTCGTCATCAGTATTCG
mhpE2	CAATCCATTCAAGGTCGCTAT
mhpC1	TTAATGCACGAATCCTGAAA
mhpC2	CATCGGCGTAAACAAACTC
rutD1	GTGTTGATTTCGGGTCTT

rutD2	CCATCTGGGCGATACTG
cadB1	TCCCGAAAGTTTATGGTG
cadB2	GTCAACGCAAGAGTAGAAGTA
hyfD1	CCGGACGCAATGGAAG
hyfD2	CTGTGGCAAATACATCAGAAAT
ydeo1	TGATTGCGTTCTTCCGAGTT
ydeo2	TGACGGTGCCTGTTCCTG
gspK1	GCGTGAAGCCTCTGGTTG
gspK2	TAGATTTGGGCGCTCTGC
eutk1	TCTCCTGCCGTCATTCCC
eutk2	AGCCGAAGCAACCCGTAA
cspA1	CTTCGGCTTCATCACTC
cspA2	TTACAGGCTGGTTACGT
ybav11	GCACTGCTCATTACCCTGTC
ybav22	GCTTTGGTTTCTACCGTTGT
ftsZ1	CTGGAAGGTGCAGACATGGT
ftsZ2	GCAACGGTCAGGATACCCAA
ftsK1	GGCTTACACCATTCCCGTCA
ftsK2	GCAGAGCAGCGCAATAGTTC
minD1	GGTTCACCCTGGTTAGAGGC
minD2	GGCATTCTGGCGTCGAAATC
pgk1	TCGTCAACCAGGTCAGCTTC
pgk2	TCGCTGACCAGCTGATTGTT
eno1	GGTGAACGCTTTGTTGCCTT
eno2	GGCATGAACACTGCTGTTGG
acnB1	GAAGCCAAATCCCCTCTGCT
acnB2	AACATCAGCAGCGTGTGAGA
mdh1	AGAGTGACCGCCAATAACCG
mdh2	CGGCATCGTGAAAAACCTGG
pgm1	TGCGACTTCCGCACAAAAAG
pgm2	AGACCGCCAATAGAAGCACC
pgi1	TCCATCCTGCTGCGTGAAAT
pgi2	GAGCTATCGTGGCTGCTGAT
galU1	CGGATATTTGGCCGTTGCTG
galU2	GCAGTCATGGCTCTTCCCTT
gyra1	ACGCGACTTGGTTGGGTATT
gyra2	TTCTCTGATCGTACCGCGTG
16s1	GATGACCAGCCACACTGGAA
16s2	GGAGTTAGCCGGTGCTTCTT

Table S3. The DEGs we selected for overexpression and their function

Locus	gene	log(fold change)	function
HO397_11705	*eutK*	-1.135	putative structural protein involving in ethanolamine utilization
HO397_14310	*glcE*	-1.781	putative FAD-binding subunit of glycolate dehydrogenase
HO397_01490	*prpR*	-1.139	propionate regulator involving in catabolism of propionate
HO397_20010	*soxR*	-0.615	"superoxide response protein", negatively autoregulated and controls the transcription of the regulon involved in defense against superoxide
